# Two consecutive intrauterine pregnancies following transperitoneal ovum migration

**DOI:** 10.1590/S1516-31802011000400012

**Published:** 2011-05-05

**Authors:** Adriano Barreto Nogueira, Ariel Barreto Nogueira, Fernanda Regina Gemi

**Affiliations:** I MD. Intensivist and neurosurgeon, Lecel Clínica Médica, São Paulo, Brazil.; II IIMD. General practitioner, Lecel Clínica Médica, São Paulo, Brazil.; III MD. Obstetrician and postgraduate student (sensu lato), Obstetrics and Gynecology Group, Hospital Beneficência Portuguesa, São Paulo, Brazil.

**Keywords:** Pregnancy, ectopic, Ultrasonography, Prenatal care, Hysterosalpingography, Infertility, Gravidez ectópica, Ultrassonografia, Cuidado pré-natal, Histerossalpingografia, Infertilidade

## Abstract

**CONTEXT:**

Transperitoneal migration is a mechanism for oocyte retrieval that is generally demonstrated in certain cases of ectopic pregnancy. However, the association between these two conditions is debatable. The rare occasions on which intrauterine pregnancy following transperitoneal migration can be documented are an opportunity for studying this topic.

**CASE REPORT:**

We report the case of a female with a history of salpingectomy due to an ectopic pregnancy at 31 years of age. Two subsequent pregnancies were intrauterine. In both of them, ultrasound revealed that the corpus luteum was located in the ovary ipsilateral to the salpingectomy.

**CONCLUSION:**

To our knowledge, this is the first reported case of two intrauterine pregnancies following transperitoneal migration, carried to term, and resulting in the delivery of two healthy children. The clinical and physiological implications are discussed.

## RESUMO

**CONTEXTO:**

A migração transperitoneal é um mecanismo de captação do oócito demonstrado, em geral, em determinados casos de gravidez ectópica. No entanto, a associação entre ambas é discutível. As raras ocasiões em que uma gestação intrauterina após migração transperitoneal pode ser documentada são uma oportunidade para o estudo deste tópico.

**RELATO DE CASO:**

Relatamos o caso de uma mulher com salpingectomia aos 31 anos, por gravidez ectópica. Duas gestações subsequentes foram intrauterinas. Em ambas, ultrassonografia evidenciou presença do corpo lúteo em ovário do mesmo lado da salpingectomia.

**CONCLUSÃO:**

No nosso conhecimento, trata-se do primeiro caso relatado com duas gestações intrauterinas após migração transperitoneal, terminadas com o nascimento de duas crianças saudáveis. As implicações clínicas e fisiológicas são discutidas.

## INTRODUCTION

The mechanism of oocyte retrieval in humans is not completely understood. Direct observation of this mechanism through hydrolaparoscopy has shown that the fallopian tube on the side of ovulation plays a key role in oocyte retrieval.^1,2^ Another possible mechanism, transperitoneal migration, has been reported.^3-16^ There is some difficulty in determining whether transperitoneal migration exists and how frequently it might occur, because of the limited number of clinical conditions under which it might be detected.

In this study, we report the case of a female with a history of right salpingectomy due to an ectopic pregnancy. In two subsequent intrauterine pregnancies, an ultrasound examination was performed within the first weeks, and it revealed that ovulation had taken place on the side ipsilateral to the salpingectomy. The reporting of cases such as this one provides data relating to the physiology and physiopathology of human reproduction, and may have clinical implications for the treatment of certain cases of subfertility.

## CASE REPORT

**Figure 1.**
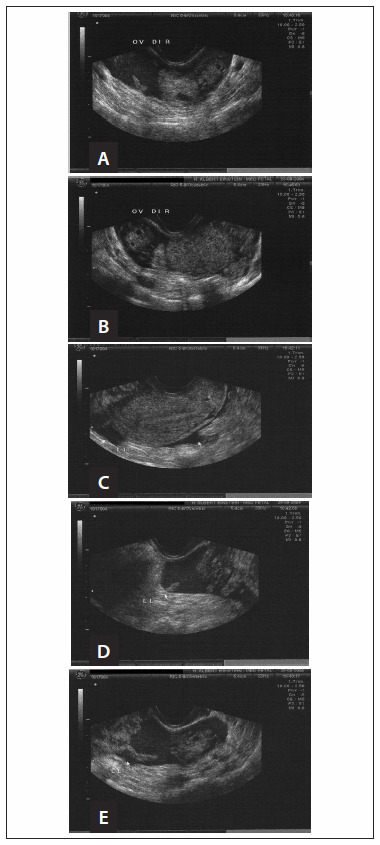
Transvaginal ultrasound on the ectopic pregnancy. Left (A and B): Right ovarian and paraovarian regions. Examination performed due to a late menstrual period, vaginal bleeding and abdominal pain. Image suggestive of tubal pregnancy. Right (C, D e E): Image suggestive of free-flowing fluid (identified as LL) in the pouch of Douglas, as confirmed by the large quantity of blood observed intraoperatively.

In September 2004, a 31-year-old female presented with vaginal bleeding. Her last menstruation had occurred 50 days earlier, when attempts to conceive had begun. During the prenatal investigation, the patient had been diagnosed with irregular menstrual periods and asymptomatic hypothyroidism. Thyroxine (50 µg/day) had been prescribed to regulate the levels of thyroid stimulating hormone. Because the bleeding persisted for longer than normal and was accompanied by abdominal pain, the patient sought medical assistance, at a private clinic in São Paulo, Brazil. Ultrasound revealed an echogenic image (probably a clot or dilated tube), measuring 25 mm × 21 mm × 21 mm, with a 8 mm anechoic image (probably a gestational sac) in the right paraovarian region, as well as an image suggestive of a large quantity of blood in the pouch of Douglas (Figure 1). The serum level of beta-human chorionic gonadotropin (β-HCG) was high (2,300 IU/l). The patient underwent right salpingectomy. The procedure was uneventful and the patient was discharged on the second postoperative day. Anatomopathological examination confirmed the diagnosis of ectopic pregnancy.

**Figure 2.**
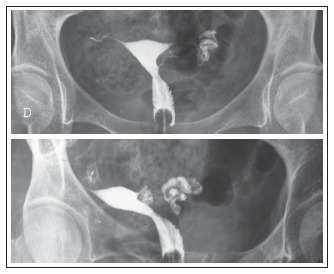
Hysterosalpingography. Examination performed during the late postoperative period following right salpingectomy. The right fallopian tube is seen as far as its middle third, the left fallopian tube is seen in its entirety, and the contrast medium can be seen to have reached the peritoneal cavity.

In December 2004, the patient began new attempts to conceive. Because the attempts were unsuccessful and her menstrual periods were irregular, the patient was started on oral progesterone replacement therapy in June 2005. At this time, the couple was also examined to determine the cause of the infertility. Hysterosalpingography showed the presence of the right fallopian tube as far as its middle third and a patent left fallopian tube, with normal diameter and preserved mucosal folds. There was normal dispersion of the contrast medium within the peritoneal cavity (Figure 2).

**Figure 3.**
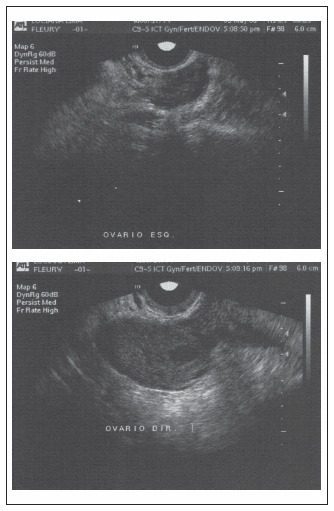
Transvaginal ultrasound on the first intrauterine pregnancy. The right ovary (image on the right) contains a cyst, which is probably the corpus luteum. No cysts are observed in the left ovary (image on the left).

After attempting to conceive for 16 months, the couple was referred to an infertility clinic. *In vitro* fertilization was scheduled for the following cycle. However, the couple conceived during that same cycle. The pregnancy was diagnosed through delayed menstruation and a β-HCG level of 7,312 IU/l. Because of the high risk of another ectopic pregnancy, an ultrasound examination was requested. Intrauterine pregnancy was confirmed, and the estimated gestational age was 6 weeks. The right ovary was enlarged and contained a cyst of 2 cm in diameter, which was probably the corpus luteum (Figure 3). The patient went into labor at gestational week 40. However, due to labor dystocia, a Cesarean section was performed. A healthy boy, weighing 3,775 g, was delivered.

**Figure 4.**
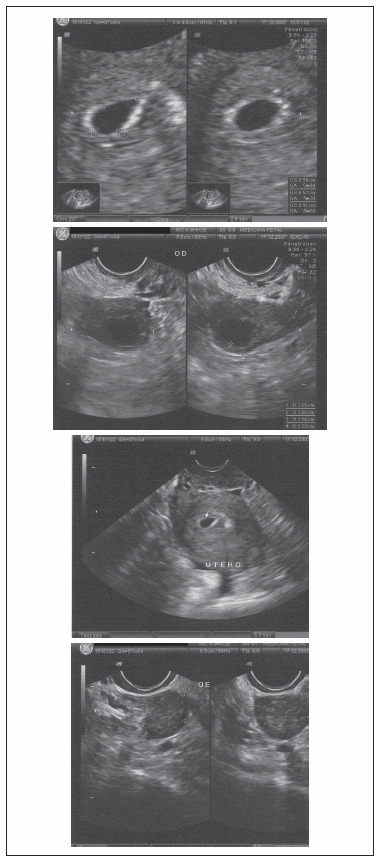
Transvaginal ultrasound of the second intrauterine pregnancy. Left upper quadrant: magnified view of the gestational sac. Right upper quadrant: gestational sac located in the uterus. Left lower quadrant: right ovary with an image suggestive of the corpus luteum. Right lower quadrant: left ovary of normal size.

The patient resumed menstruation at seven months after delivery. Two months after the return of menses, the couple again began attempts to conceive. Conception occurred four months later, while the patient was still breastfeeding. When the pregnancy was diagnosed, the serum level of β-HCG was 1,719 IU/l. For the same reasons as in the first pregnancy, an ultrasound examination was requested (Figure 4), and intrauterine pregnancy was again confirmed. The right ovary was larger than the left and was seen to contain a simple cyst, of 1.2 cm in diameter. At 39 weeks of gestation, a healthy boy, weighing 3,810 g, was born via vaginal delivery.

## DISCUSSION

Oocyte retrieval by the fimbriae is a stage of human reproduction rarely observed clinically. One technique that allows observation of this process under physiological conditions is transvaginal hydrolaparoscopy.^1,2^ Through transvaginal hydrolaparoscopy, vascular congestion causing erection of the fimbriae on the side of ovulation can be observed. The fimbriae move closer to the caudal pole of the ovary responsible for the release of the cumulus oophorus, and movements relating to the vascular congestion aid in the retrieval of the oocyte.

During oocyte retrieval, it has been reported that the contralateral fallopian tube remains unaltered.^1,2^ However, the authors of that report, who also proposed transvaginal hydrolaparoscopy, themselves speculated about the potential limitations of their method. The changes observed in the fallopian tube on the side of ovulation suggest that the ovary may participate in oocyte retrieval. The control exerted by the ovary might be due to release of β-estradiol, which has a vasodilatory effect. During hydrolaparoscopy, it is possible that the saline solution used causes dilution of β-estradiol, which would explain the lack of changes in the contralateral fallopian tube. In addition, only a small number of cases have been investigated using transvaginal hydrolaparoscopy.

Experimental studies in animals and clinical observations in humans have provided evidence of the existence of external migration of spermatozoa or ova. Intrauterine pregnancy has been diagnosed in rabbits subjected to oophorectomy and contralateral salpingectomy.^12^ There is also clinical evidence of another physiological mechanism for oocyte retrieval,^16^ through which the oocyte is released into the pouch of Douglas, with an equal chance of being retrieved by either fallopian tube.

Although oocyte retrieval is not fully understood, clinical evidence suggestive of transperitoneal migration has been reported.^3-16^ In such cases, fallopian tube obstruction, due to congenital malformations or acquired conditions, has been observed. Another condition under which transperitoneal migration has been documented is a non-communicating rudimentary uterine horn pregnancy, associated with a contralateral corpus luteum.^5^

Transperitoneal migration is considered to be a contributory factor for ectopic pregnancy. This assertion is based on the fact that the corpus luteum is found to be on the contralateral side in a high proportion (15-20%) of the cases of ectopic pregnancy.^7,13,15^ Furthermore, the supposed association between infertility and transperitoneal migration has led some authors to suggest that oophorectomy, ipsilateral to the salpingectomy, should be performed in certain cases of tubal pregnancy.^13^

On the other hand, one case series^14^ and one meta-analysis^5^ estimated that the incidence of transperitoneal migration is higher than had been suspected. Considering the relatively low incidence of ectopic pregnancy, these studies concluded that the two conditions were unrelated. However, the conclusions in these studies were not based on data from clinically and radiologically documented intrauterine pregnancies following transperitoneal migration.

To examine the concept that transperitoneal migration may be associated with intrauterine pregnancy, a literature review was conducted. Medline, Embase, Cochrane Library, Lilacs and Scielo were accessed for original articles (Table 1). The initial search strategy included the key words "transperitoneal migration" or "external migration" ("migração transperitoneal" or "migração externa" in Portuguese). The texts and references of the selected articles were used for a final search. We found 11 reports (10 case reports and 1 case series) of intrauterine pregnancies associated with transperitoneal migration (Table 2).^6,8,10,12^ A systematic review is included in one of these articles.^12^ Only Motta et al.^8^ documented more than one intrauterine pregnancy (with two abortions and one that ended at term). We did not find any reports of more than one successful pregnancy that might resemble the present case.

**Table 1.** Strategy for literature review on intrauterine pregnancy following transperitoneal migration of ova or spermatozoa

Source	Search strategy	Result: ectopic and intrauterine pregnancies	Intrauterine pregnancies only
Medline	"transperitoneal migration" or "external migration"	15 case reports 2 case series	10 case reports 1 case series*
Embase	"transperitoneal migration" or "external migration"	16 case reports 2 case series	
Cochrane Library, Lilacs an SciElo	0	0	0

* transperitoneal migration associated with any type of pregnancy, with only one intrauterine pregnancy.

**Table 2.** Reported cases of intrauterine pregnancies following transperitoneal ovum migration (1985-2009)

Reference	Number of cases/pregnancies	Main characteristics	Methods for evidence of transperitoneal migration	Outcome
Ombelet et al.^6^	1/1	Unicornuate uterus; ectopic ovary	HS, US, MRI, laparoscopy and hysteroscopy	Cesarean section
Motta et al.^8^	1/3	Left OE and right SE for ectopic pregnancy and ovarian cyst	HS, US	1^st^) cesarean section; 2^nd^) spontaneous abortion; 3^rd^) induced abortion
Ben-Nun et al.^10^	1/1	Right SE for ruptured tubal pregnancy	Laparoscopy: left ovary absent	Vaginal delivery at term
Wheeler and Dodson^12^*	8/8	Ovarian hematoma; congenital absence of tube; tubal pregnancy	Operative procedures (OE and SE); US	Term infants in 8 pregnancies

* case report and a review of intrauterine pregnancies following transperitoneal migration (up to 1985; see Table 1 of this reference); HS = hysterosalpingography, MRI = magnetic resonance imaging; OE = oophorectomy; SE = salpingectomy; US = ultrasound.

## CONCLUSION

The case presented here suggests that transperitoneal migration in humans may be more common than has been presumed. The possibility that an intrauterine pregnancy can follow such migration should be taken into consideration.
